# Supplementary material for: Could Reducing Body Fatness Reduce the Risk of Aggressive Prostate Cancer via the Insulin Signalling Pathway? A Systematic Review of the Mechanistic Pathway
Source: Metabolites. 2021 Oct 23;11(11):726. doi: 10.3390/metabo11110726 (PMC8625823; doi:10.3390/metabo11110726)
Supplement: Supplementary file 1 [file metabolites-11-00726-s001.zip › metabolites-1414688-supplementary.pdf]

Supplementary Material

Supplementary Results Tables

Table S1 -Reasons for exclusion for the Body-fatness - Insulin set of studies (Randomized controlled trials)

**Study ID**	**PMID**	**Reasons for exclusion**
Alsubheen et al., 2017	29198194	Study design: no randomization / no allocation concealment
Dengel et al., 1998	9880120	Study design: no randomization / no allocation concealment
McAllister et al., 2020	31955013	Study design: two intervention groups and no control group
Kauka et al., 2003	12805389	No relevant results available for biomarkers and surrogate indices of the insulin signalling pathway and insulin resistance
Johnson et al., 2016	26324180	No separate analysis for males
Wright et al., 2013	23775525	Participants were men with newly diagnosed prostate cancer cases at baseline
Chan et al., 2008	18837799	No eligible participants: men with MetS
Jasobs et al., 2009	19116328	No eligible participants: 50% of participants had MetS
Moro et al., 2016	27737674	No eligible participants: resistance-trained males for at least 5 years
Ng et al., 2009	19456294	No eligible participants: men with MetS
Tanaka et al., 2014	25744418	No eligible participants: men with MetS

Table S2: Risk of bias assessment for body fatness-insulin studies per domain and overall using the Robins-I tool (REFERENCE)

Study ID	Weight	D1	D2	D3	D4	D5	Overall
Teng et al., 2013	1	!	+	+	+	+	!
Guo et al., 2018	1	!	-	!	+	+	-
Ross et al., 2000	1	+	!	+	+	+	!
Pritchard et al., 2002	1	+	-	!	+	+	-
Katzel et al., 1995	1	+	!	+	+	+	!
Joris et al., 2016	1	+	-	+	+	+	-
Alves et al., 2014, A	1	+	-	!	+	+	-
Alves et al., 2014, B	1	+	-	!	+	+	-

+	Low risk
!	Some concerns
-	High risk
D1	Randomisation process
D2	Deviations from the intended interventions
D3	Missing outcome data
D4	Measurement of the outcome
D5	Selection of the reported result

Table S3: Converted and calculated data (Body-Fatness - Insulin set of studies)

Study characteristics			Control (pre-treatment)		Control (post-treatment)		Control (pre-post change)		Intervention (pre-treatment)		Intervention (post-treatment)		Intervention (pre-post cange)		Pre-post change (intervention vs. control)		
Study ID	Sample size (completed trial)	Exposure unit	Mean	SD	Mean	SD	Mean	SD	Mean	SD	Mean	SD	Mean	SD	Mean	SD	P-value
**Fasting glucose**																	
Joris et al., 2016	49 (23 / 26)	mmol/L	5.75	0.53	5.75	0.62	0.00	0.57	5.49	0.37	5.29	0.34	-0.20	0.37	-0.20	0.49	0.15
Teng et al., 2013	56 (28 / 28)	mmol/l	6.00	0.70	6.50	1.40	0.50	1.13	5.90	0.50	6.10	0.60	0.20	0.56	-0.30	0.89	0.21
Guo et al., 2018	80 (42 / 38)	mmol/L	5.40	1.10	5.40	1.20	0.00	1.14	5.80	1.20	5.50	1.50	-0.30	1.36	-0.30	1.26	0.29
Ross et al., 2000	22 (14 / 8)	mmol/L	5.00	0.70	4.90	0.70*	-0.10	0.68	5.30	0.50	5.10	NA	-0.20	0.50	-0.10	0.56	0.72
Alves et al., 2014, A	39 (21 / 18)	mmol/L	4.81	0.72	NA	NA	0.06	0.27	4.73	0.59	4.98	NA	0.24	0.24	0.18	0.27	**0.03**
Alves et al., 2014, B	37 (19 / 18)	mmol/L	4.81	0.72	NA	NA	0.06	0.27	4.82	0.51	4.98	NA	0.16	0.37	0.09	0.32	0.38
Katzel et al., 1995	62 (44 / 18)	mmol/L	5.77	0.72	NA	NA	NA	NA	5.66	0.73	NA	NA	2% decrease	NA	NA	NA	NA
**Fasting insulin**																	
Joris et al., 2016	49 (23 / 26)	pmol/L	79.86	38.89	81.25	36.81	1.39	37.49	77.78	34.72	51.39	22.92	-26.39	32.00	-27.78	37.39	**0.01**
Pritchard et al., 2002	24 (12 / 12)	pmol/L	71.53	32.64	63.89	27.08	-7.64	26.65	81.25	40.28	66.67	26.39	-14.58	34.69	-6.94	30.46	0.59
Ross et al., 2000	22 (14 / 8)	pmol/L	52.00	29.00	54.00	29.00*	2.00	28.04	56.00	21.00	46.00	NA	-10.50	27.60	-12.50	27.77	0.33
Alves et al., 2014, A	39 (21 / 18)	pmol/L	57.64	28.47	NA	NA	-2.78	25.21	42.36	17.36	55.07	NA	12.71	20.28	15.49	23.70	**0.04**
Alves et al., 2014, B	37 (19 / 18)	pmol/L	57.64	28.47	NA	NA	-2.78	25.21	51.39	22.22	51.11	NA	-0.28	13.82	2.50	19.93	0.71
Katzel et al., 1995	62 (44 / 18)	mmol/L	NA	NA	NA	NA	NA	NA	NA	NA	NA	NA	18% decrease	NA	NA	NA	NA
**HOMA-IR**																	
Joris et al., 2016	49 (23 / 26)	-	2.90	1.40	2.96	1.45	0.06	1.41	2.64	1.21	1.67	0.79	-0.97	1.12	-1.03	1.37	**0.01**
Alves et al., 2014, A	39 (21 / 18)	-	1.80	1.00	NA	NA	-0.04	0.81	1.30	0.60	1.82	NA	0.52	0.77	0.56	0.83	**0.03**
Alves et al., 2014, B	37 (19 / 18)	-	1.80	1.00	NA	NA	-0.04	0.81	1.60	0.80	1.62	NA	0.02	0.48	0.06	0.65	0.79
**C-peptide**																	
Joris et al., 2016	49 (23 / 26)	ng/mL	1.75	0.69	1.76	0.63	0.01	0.65	1.59	0.59	1.25	0.46	-0.34	0.55	-0.35	0.63	**0.05**
**OGTT glucose (2-hour)**																	
Ross et al., 2000	22 (14 / 8)	mmol/L * 2 h	29.00	6.80	28.80	6.80*	-0.20	6.57	29.70	5.90	26.80	NA	-2.90	5.20	-2.70	5.74	0.34
Katzel et al., 1995	62 (44 / 18)	mmol/L	8.60	0.72	NA	NA	11% increase	NA	8.32	2.59	NA	NA	8% decrease	NA	NA	NA	NA
**OGTT insulin (2-hour)**																	
Ross et al., 2000	22 (14 / 8)	pmol/L * 2 h	1457.00	1200.00	1465.00	1200.00*	8.00	1159.32	1508.00	1122.00	1114.00	NA	-393.00	780.00	-401.00	929.31	0.40
Katzel et al., 1995	62 (44 / 18)	mmol/L	NA	NA	NA	NA	12% increase	NA	NA	NA	NA	NA	26% decrease	NA	NA	NA	NA
**Glucose disposal rate**																	
Ross et al., 2000	22 (14 / 8)	mg/kg muscle per minute	15.40	6.00	14.40	6.00*	-1.00	5.82	13.00	6.10	18.60	NA	5.60	3.40	6.60	5.39	**0.02**
**Glucose disposal (Oxidative fraction)**																	

Ross et al., 2000	22 (14 / 8)	mg/kg muscle per minute	3.40	1.20	2.90	1.20*	-0.50	1.19	2.70	1.50	2.80	1.50*	0.10	1.47	0.60	1.38	0.31
**Glucose disposal (Nonoxidative fraction)**																	
Ross et al., 2000	22 (14 / 8)	mg/kg muscle per minute	12.00	5.90	11.70	5.90*	-0.30	5.70	11.50	5.40	17.10	5.40*	5.60	6.02	5.90	6.46	**0.04**

Shaded boxes represent figures calculated by us according to the methods described in the statistical analysis section above.

Table S4 -Reasons for exclusion for the Insulin-PCa set of studies

Study ID	PMID	Study Design	Population-based / Hospital-based	Reasons for exclusion
Stocks et al., 2007	17278097	Case-control study nested in a prospective cohort	Population	No BMI adjustment
Arthur et al., 2019	30421156	Prospective cohort	Population	No BMI adjustment
Inoue et al., 2009	19491612	Prospective cohort	Population	No BMI adjustment
Hubbard et al., 2004	14972466	Prospective cohort	Population	No BMI adjustment
Travier et al., 2007	17693655	Prospective cohort	Population	No BMI adjustment
Tande et al., 2006	16968859	Multicenter prospective cohort	Population	No BMI adjustment
Murtola et al., 2018	29563633	Prospective cohort	Population	No BMI adjustment
Jee et al., 2005	15644546	Propsective cohort	Population	No BMI adjustment
Murtola et al., 2019	30679762	Prospective cohort	Population	No BMI adjustment
Kiyabu et al., 2018	28362652	Case-control study nested in a prospective cohort	Hospital	Exposure measured less than 2 years prior to outcome or mean / median follow-up was less than 5 years
Stattin et al., 2000	11106682	Case-control study nested in a prospective cohort	Population	Exposure measured less than 2 years prior to outcome or mean / median follow-up was less than 5 years

Stevens et al., 2014	24585409	Case-control study nested in a prospective cohort	Population	Exposure measured less than 2 years prior to outcome or mean / median follow-up was less than 5 years
Goto et al., 2016	26547128	Propsective cohort	Population	Exposure measured less than 2 years prior to outcome or mean / median follow-up was less than 5 years
Joshu et al., 2012	22161730	Propsective cohort	Population	Exposure measured less than 2 years prior to outcome or mean / median follow-up was less than 5 years
Kim et al., 2018	29268567	Prospective cohort	Population	Exposure measured less than 2 years prior to outcome or mean / median follow-up was less than 5 years
Parekh et al., 2013	24064521	Prospective cohort	Population	Exposure measured less than 2 years prior to outcome or mean / median follow-up was less than 5 years
Grundmark et al., 2010	20647401	Propsective cohort	Population	Includes non-eligible participants: exposure of interest was MetS
Nguyen et al., 2018	28692586	Randomized, double-blind, placebo-controlled trial	Hospital (PCa patients scheduled to undergo radical prostatectomy)	No relevant data

Table S5: Risk of bias assessment per domain and overall for the insulin-prostate cancer study, using the Robins-E risk of bias tool

Study ID	Exposure	Outcome	D1	D2	D3	D4	D5	D6	D7	Overall
Lai GY (2010)	C-peptide	PCa total	!	+	+	+	+	+	+	!
Lai GY (2014)	C-peptide	PCa total	!	+	+	+	+	+	+	!
Lai GY (2010)	C-peptide	PCa, localised	!	+	+	+	+	+	+	!
Lai GY (2014)	C-peptide	PCa, localised	!	+	+	+	+	+	+	!
Lai GY (2010)	C-peptide	PCa, advanced	!	+	+	+	+	+	+	!
Lai GY (2014)	C-peptide	PCa, advanced	!	+	+	+	+	+	+	!
Lai GY (2010)	C-peptide	PCa, low-grade	!	+	+	+	+	+	+	!
Lai GY (2014)	C-peptide	PCa, low-grade	!	+	+	+	+	+	+	!
Lai GY (2010)	C-peptide	PCa, high-grade	!	+	+	+	+	+	+	!
Lai GY (2014)	C-peptide	PCa, high-grade	!	+	+	+	+	+	+	!
Albanes D (2009)	Fasting glucose	PCa total	!	+	+	+	+	+	+	!
Dickerman BA (2018)	Fasting glucose	PCa total	!	+	+	+	!	+	+	!
Marrone MT (2019)	Fasting glucose	PCa total	!	+	+	+	+	+	+	!
Dickerman BA (2018)	Fasting glucose	PCa, advanced	!	+	+	+	-	+	+	-
Marrone MT (2019)	Fasting glucose	PCa, advanced	!	+	+	+	!	+	+	!
Dickerman BA (2018)	Fasting glucose	PCa mortality	!	+	+	+	!	+	+	!
Marrone MT (2019)	Fasting glucose	PCa mortality	!	+	+	+	!	+	+	!
Dickerman BA (2018)	Fasting glucose	PCa, high-grade	!	+	+	+	-	+	+	-
Albanes D (2009)	Fasting insulin	PCa total	!	+	+	+	+	+	+	!
Albanes D (2009)	Fasting insulin	PCa, localised	!	+	+	+	+	+	+	!
Albanes D (2009)	Fasting insulin	PCa, advanced	!	+	+	+	+	+	+	!
Marrone MT (2019)	HbA1c (%)	PCa total	!	+	+	+	+	+	+	!
Marrone MT (2019)	HbA1c (%)	PCa, advanced	!	+	+	+	!	+	+	!
Marrone MT (2019)	HbA1c (%)	PCa mortality	!	+	+	+	!	+	+	!
Darbinian JA (2008)	Glucose tolerance	PCa total	!	+	+	+	!	+	+	!
Darbinian JA (2008)	Glucose tolerance	PCa, localised	!	+	+	+	!	+	+	!
Darbinian JA (2008)	Glucose tolerance	PCa, regional (stages 2-5), distant (stage 7)	!	+	+	+	!	+	+	!
Albanes D (2009)	HOMA-IR	PCa total	!	+	+	+	+	+	+	!
Albanes D (2009)	Molar ratio of insulin to	PCa total	!	+	+	+	+	+	+	!

+	Low risk
+	Low risk except for concerns of uncontrolled confounding
!	Some concerns
-	High risk
-	Very high risk
D1	Risk of bias due to confounding
D2	Risk of bias arising from measurement of the exposure
D3	Risk of bias in selection of participants into the study (or into the analysis)
D4	Risk of bias due to post-exposure interventions
D5	Risk of bias due to missing data
D6	Risk of bias arising from measurement of outcomes
D7	Risk of bias in selection of the reported result

Table S6: Descriptive results as reported by the studies (Insulin - PCa set of studies)

Author (date)	Study design	Outcomes assessed	Effect	Numerical result	Relationship analysed	P-value for trend	Risk of bias assessment
**C-peptide**							
Lai GY (2014)	Case-control studies nested in a prospective cohort	PCa total	Increased risk	OR=1.22 (95% CI: 0.98 to 1.50)	Q2 vs. Q1*	0.99	Some concerns
				OR=1.07 (95% CI: 0.85 to 1.35)	Q3 vs. Q1*		
				OR=1.05 (95% CI: 0.83 to 1.33)	Q4 vs. Q1*		
Lai GY (2010)	Case-control studies nested in a prospective cohort	PCa total	Lower risk	OR=0.83 (95% CI: 0.43 to 1.63)	Q2 vs. Q1 (849-1367 vs. <849 pmol/L)	0.04	Some concerns
				OR=0.69 (95% CI: 0.35 to 1.36)	Q3 vs. Q1 (1367-2017 vs. <849 pmol/L)		

				OR=0.40 (95% CI: 0.17 to 0.96)	Q4 vs. Q1 (≥2017 vs. <849 pmol/L)		
Lai GY (2014)	Case-control studies nested in a prospective cohort	PCa, localised	Increased risk	OR=1.03 (95% CI: 0.80 to 1.33)	Q4 vs. Q1*	0.880	Some concerns
Lai GY (2010)	Case-control studies nested in a prospective cohort	PCa, localised	Lower risk	OR=0.77 (95% CI: 0.36 to 1.66)	Q2 vs. Q1 (849-1367 vs. <849 pmol/L)	0.04	Some concerns
				OR=0.50 (95% CI: 0.24 to 1.05)	Q3 vs. Q1 (1367-2017 vs. <849 pmol/L)		
				OR=0.44 (95% CI: 0.19 to 1.03)	Q4 vs. Q1 (≥2017 vs. <849 pmol/L)		
Lai GY (2014)	Case-control studies nested in a prospective cohort	PCa, advanced	Increased risk	OR=1.18 (95% CI: 0.69 to 2.03)	Q4 vs. Q1*	0.780	Some concerns
Lai GY (2010)	Case-control studies nested in a prospective cohort	PCa, advanced	Increased risk	OR=1.39 (95% CI: 0.47 to 4.09)	Q2 vs. Q1 (849-1367 vs. <849 pmol/L)	0.39	Some concerns
				OR=1.50 (95% CI: 0.47 to 4.82)	Q3 vs. Q1 (1367-2017 vs. <849 pmol/L)		
				OR=1.83 (95% CI: 0.50 to 6.78)	Q4 vs. Q1 (≥2017 vs. <849 pmol/L)		
Lai GY (2014)	Case-control studies nested in a prospective cohort	PCa, low-grade	Lower risk	OR=0.98 (95% CI: 0.74 to 1.30)	Q4 vs. Q1*	0.500	Some concerns
Lai GY (2010)	Case-control studies nested in a prospective cohort	PCa, low-grade	Inconclusive	OR=1.13 (95% CI: 0.56 to 2.31)	Q2 vs. Q1 (849-1367 vs. <849 pmol/L)	0.28	Some concerns
				OR=0.92 (95% CI: 0.44 to 1.91)	Q3 vs. Q1 (1367-2017 vs. <849 pmol/L)		
				OR=0.68 (95% CI: 0.30 to 1.54)	Q4 vs. Q1 (≥2017 vs. <849 pmol/L)		
Lai GY (2014)	Case-control studies nested in a prospective cohort	PCa, high-grade	Increased risk	OR=1.20 (95% CI: 0.87 to 1.66)	Q4 vs. Q1*	0.280	Some concerns
Lai GY (2010)	Case-control studies nested in a prospective cohort	PCa, high-grade	Lower risk	OR=0.32 (95% CI: 0.11 to 0.97)	Q2 vs. Q1 (849-1367	0.15	Some concerns

					vs. <849 pmol/L)		
				OR=0.57 (95% CI: 0.18 to 1.83)	Q3 vs. Q1 (1367-2017 vs. <849 pmol/L)		
				OR=0.57 (95% CI: 0.18 to 1.83)	Q4 vs. Q1 (≥2017 vs. <849 pmol/L)		
**Fasting glucose**							
Albanes D (2009)	Case-control studies nested in a prospective cohort	PCa total	Inconclusive	OR=1.33 (95% CI: 0.72 to 2.48)	Q2 vs. Q1 (93-99 vs. ≤93 mg/dL)	0.38	Some concerns
				OR=0.92 (95% CI: 0.46 to 1.86)	Q3 vs. Q1 (99-107 vs. ≤93 mg/dL)		
				OR=1.43 (95% CI: 0.76 to 2.68)	Q4 vs. Q1 (>107 vs. ≤93 mg/dL)		
Dickerman BA (2018)	Prospective cohorts	PCa total	Lower risk	HR=0.70 (95% CI: 0.48 to 1.02)	Cat2 vs. Cat1 (100-126 vs. <100 mg/dL and no history of diabetes)	>0.05	Some concerns
Marrone MT (2019)	Prospective cohorts	PCa total	Lower risk	HR=0.97 (95% CI: 0.81 to 1.15)	Cat2 vs. Cat1 (≥ 5.6 vs. 3.1-5.6 mmol/L)	>0.05	Some concerns
Dickerman BA (2018)	Prospective cohorts	PCa, advanced	Lower risk	HR=0.74 (95% CI: 0.40 to 1.35)	Cat2 vs. Cat1 (100-126 vs. <100 mg/dL and no history of diabetes)	>0.05	High risk
Marrone MT (2019)	Prospective cohorts	PCa, advanced	Increased risk	HR=1.98 (95% CI: 1.05 to 3.72)	Cat2 vs. Cat1 (≥ 5.6 vs. 3.1-5.6 mmol/L)	**<0.05**	Some concerns
Dickerman BA (2018)	Prospective cohorts	PCa mortality	Lower risk	HR=0.87 (95% CI: 0.32 to 2.36)	Cat2 vs. Cat1 (100-126 vs. <100 mg/dL and no history of diabetes)	>0.05	Some concerns
Marrone MT (2019)	Prospective cohorts	PCa mortality	Increased risk	HR=1.83 (95% CI: 1.00 to 3.37)	Cat2 vs. Cat1 (≥ 5.6	**<0.05**	Some concerns

					vs. 3.1-5.6 mmol/L)		
Dickerman BA (2018)	Prospective cohorts	PCa, high-grade	Lower risk	HR=0.79 (95% CI: 0.42 to 1.49)	Cat2 vs. Cat1 (100-126 vs. <100 mg/dL and no history of diabetes)	>0.05	High risk
**Fasting insulin**							
Albanes D (2009)	Case-control studies nested in a prospective cohort	PCa total	Increased risk	OR=1.50 (95% CI: 0.75 to 3.03)	Q2 vs. Q1 (2.75-4.10 vs. ≤2.75 µU/mL)	0.02	Some concerns
				OR=1.75 (95% CI: 0.86 to 3.56)	Q3 vs. Q1 (4.10-6.10 vs. ≤2.75 µU/mL)		
				OR=2.55 (95% CI: 1.18 to 5.51)	Q4 vs. Q1 (>6.10 vs. ≤2.75 µU/mL)		
Albanes D (2009)	Case-control studies nested in a prospective cohort	PCa, localised	Increased risk	OR=1.62 (95% CI: 0.68 to 3.90)	Q2 vs. Q1 (2.75-4.10 vs. ≤2.75 µU/mL)	0.02	Some concerns
				OR=2.36 (95% CI: 1.00 to 5.56)	Q3 vs. Q1 (4.10-6.10 vs. ≤2.75 µU/mL)		
				OR=3.19 (95% CI: 1.25 to 8.13)	Q4 vs. Q1 (>6.10 vs. ≤2.75 µU/mL)		
Albanes D (2009)	Case-control studies nested in a prospective cohort	PCa, advanced	Inconclusive	OR=1.38 (95% CI: 0.49 to 3.91)	Q2 vs. Q1 (2.75-4.10 vs. ≤2.75 µU/mL)	0.57	Some concerns
				OR=0.98 (95% CI: 0.30 to 3.21)	Q3 vs. Q1 (4.10-6.10 vs. ≤2.75 µU/mL)		
				OR=1.53 (95% CI: 0.44 to 5.35)	Q4 vs. Q1 (>6.10 vs. ≤2.75 µU/mL)		
**HbA1c (%)**							

Marrone MT (2019)	Prospective cohorts	PCa total	Lower risk	HR=0.98 (95% CI: 0.81 to 1.18)	Cat3 vs. Cat2 (> 5.6 vs. 5.0-5.6 %)	>0.05	Some concerns
Marrone MT (2019)	Prospective cohorts	PCa, advanced	Increased risk	HR=1.40 (95% CI: 0.77 to 2.54)	Cat3 vs. Cat2 (> 5.6 vs. 5.0-5.6 %)	>0.05	Some concerns
Marrone MT (2019)	Prospective cohorts	PCa mortality	Increased risk	HR=1.32 (95% CI: 1.74 to 2.36)	Cat3 vs. Cat2 (> 5.6 vs. 5.0-5.6 %)	**<0.05**	Some concerns
**Glucose tolerance**							
Darbinian JA (2008)	Prospective cohorts	PCa total	Lower risk	RR=0.89 (95% CI: 0.78 to 1.02)	Cat2 vs. Cat1 (140-159 vs. <140 mg/dL)	0.005	Some concerns
				RR=0.95 (95% CI: 0.85 to 1.06)	Cat3 vs. Cat1 (160-199 vs. <140 mg/dL)		
				RR=0.87 (95% CI: 0.77 to 0.97)	Cat4 vs. Cat1 (≥200 vs. <140 mg/dL)		
Darbinian JA (2008)	Prospective cohorts	PCa, localised	Lower risk	RR=0.90 (95% CI: 0.77 to 1.06)	Cat2 vs. Cat1 (140-159 vs. <140 mg/dL)	0.02	Some concerns
				RR=0.94 (95% CI: 0.83 to 1.07)	Cat3 vs. Cat1 (160-199 vs. <140 mg/dL)		
				RR=0.87 (95% CI: 0.76 to 1.00)	Cat4 vs. Cat1 (≥200 vs. <140 mg/dL)		
Darbinian JA (2008)	Prospective cohorts	PCa, regional (stages 2-5), distant (stage 7)	Inconclusive	RR=0.86 (95% CI: 0.64 to 1.14)	Cat2 vs. Cat1 (140-159 vs. <140 mg/dL)	0.88	Some concerns
				RR=1.07 (95% CI: 0.86 to 1.33)	Cat3 vs. Cat1 (160-199 vs. <140 mg/dL)		
				RR=0.99 (95% CI: 0.78 to 1.24)	Cat4 vs. Cat1 (≥200		

					vs. <140 mg/dL)		
**HOMA-IR**							
Albanes D (2009)	Case-control studies nested in a prospective cohort	PCa total	Increased risk	OR=0.82 (95% CI: 0.40 to 1.65)	Q2 vs. Q1 (0.69-1.02 vs. ≤0.69)	0.02	Some concerns
				OR=1.17 (95% CI: 0.59 to 2.34)	Q3 vs. Q1 (1.02-1.53 vs. ≤0.69)		
				OR=2.10 (95% CI: 1.03 to 4.26)	Q4 vs. Q1 (>1.53 vs. ≤0.69)		
**Molar ratio of insulin to glucose**							
Albanes D (2009)	Case-control studies nested in a prospective cohort	PCa total	Increased risk	OR=1.11 (95% CI: 0.56 to 2.19)	Q2 vs. Q1 (0.03-0.04 vs. ≤0.03)	0.12	Some concerns
				OR=1.41 (95% CI: 0.71 to 2.78)	Q3 vs. Q1 (0.04-0.06 vs. ≤0.03)		
				OR=1.75 (95% CI: 0.83 to 3.67)	Q4 vs. Q1 (>0.06 vs. ≤0.03)		
*Cutpoints based on the distributions among controls for each batch (4 batches based on dates of diagnosis)							

Supplementary Methods

Sequential/Hierarchical approach used to identify and select studies

1. In the first instance we examined systematic reviews of randomized trials (for the body fatness – insulin signalling association), and either randomized trials or prospective observational studies (for the insulin signalling – prostate cancer association), by applying a database relevant systematic review filter to our searches. We first examined the most recent systematic review that met all the following criteria:
    - Provided a clear research question using a PICO/PECO approach that subsumes the PICO/PECO for our review.
    - Applied pre-specified eligibility criteria.
    - Took a systematic approach to the literature search, providing details of the databases searched and at least one full search strategy and had study selection and key elements of data extraction involving at least two investigators (either done independently or one independently checking the other's decisions).
    - Provided sufficient information about the included studies to allow identification of those that met the eligibility criteria for our review.

This review was then termed a 'source review'.

2. We then employed an iterative process by examining the next most recent systematic review meeting the above criteria (a further source review) and so on until the reviews we examined did not add any new studies.

Primary studies from the source reviews that themselves met the study-specific inclusion criteria for this review were extracted and used as our unit of interest.

For the insulin-PCa side of the search we performed a search to identify further primary studies from the date of the last search from the source reviews, drawing on

the search methods described in those reviews. We also searched for primary randomized trials using appropriate study search filters, as we found only found reviews of observational studies. Additionally, as limited reviews, and primary studies from these reviews, met our inclusion criteria for the body fatness-insulin search, we searched for primary randomized studies from the inception dates of the databases used to the day of the search.

Statistical analysis

Where it was reported in the paper we extracted the within groups pre-post mean difference in insulin biomarker levels from the RCTs of body fatness and insulin biomarkers. Where this was not reported a paired t-test was used to obtain the pre-post intervention mean difference (md) in biomarker levels within groups. Paired t-test is a function of the covariance, a metric rarely reported by the authors, and requires individual level data. Out of the 7 eligible studies, only one fully reported paired t-test results; mean difference (md) in biomarker levels, standard deviation of the mean difference (sddiff) and number of participants (n) for both groups (Alves et al., 2014). For the remaining studies, when mean biomarkers levels (mi), standard deviation (sdi), and number of participants (ni) pre- and post- intervention were provided, md was approximated via t-test for non-paired summary data, assuming equal variances.

In Ross et al. (2000) raw data on post-intervention standard deviation (sdi) for controls was lacking and so, we assumed equal pre- and post-intervention variances.

Between groups pre-post mean difference:

Likewise, the pre-post change between groups (intervention vs. control) was fully reported by only one study [Joris et al., 2016]. For the rest of the studies, the p-value or p-threshold from the statistical test performed [t-test, analysis of variance

(ANOVA) or analysis of covariance (ANCOVA) and the corresponding post hoc test] was provided instead. To estimate the difference between groups, t-test for non-paired summary data assuming unequal variances was applied to data reported by the authors or calculated as previously described. Where there was a lack of fully reported data, pre-post change in biomarkers levels between groups is presented as md, standard sddiff, and p-value determined by t-test using Stata version 16 (Stata Corporation, College Station, Texas, USA). Where results were fully reported, both published (supplementary table 2) and calculated results (supplementary table 3) are provided.

Conversion of fasting glucose and fasting insulin to standard units:

Fasting glucose: 1 mmol/L = 18.018 mg/dL

Fasting insulin: 1 µU/mL = 6.9444 pmol/L, 1 mU/L = 6.9444 pmol/L, 1 mmol/L = 1E+09 pmol/L

Search strategies

MEDLINE search strategy

Body fatness - Insulin signalling systematic review search

1. *body fat distribution/ or *adiposity/ or *body mass index/ or *body weight/ or *overweight/ or *obesity/ or *waist circumference/ or *waist-height ratio/ or *skinfold thickness/ or *waist-hip ratio/
2. exp *Adipose Tissue/me, pp [Metabolism, Physiopathology]
3. *body weight changes/ or *weight gain/ or *weight loss/
4. or/1-3
5. *Insulin/bl [Blood]
6. *Insulin Resistance/ or Insulin Resistance/ge [Genetics]
7. exp *Hyperinsulinism/
8. *Glycated Hemoglobin A/me [Metabolism]
9. or/5-8
10. 4 and 9
11. ((adiposity or body mass or BMI or fat or fatness or weight or overweight or over-weight or obese or obesity) adj3 (insulin* or hyperinsulin*)).ti,ab,kf.
12. ((adiposity or body mass or BMI or fat or fatness or weight or overweight or over-weight or obese or obesity) adj5 (fasting glucose or proinsulin or pro-insulin or c-peptide or HbA1c or h?emoglobin A1c or HOMA-IR or HOMA-S or HOMA or QUICKI)).ti,ab,kf.
13. (waist adj2 (hip or height) adj2 ratio? adj5 (insulin* or hyperinsulin*)).ti,ab,kf.
14. (waist adj2 (hip or height) adj2 ratio? adj5 (fasting glucose or proinsulin or pro-insulin or c-peptide or HbA1c or h?emoglobin A1c or HOMA-IR or HOMA-S or HOMA or QUICKI)).ti,ab,kf.
15. or/10-14
16. (systematic or structured or evidence or trials or studies).ti. and ((review or overview or look or examination or update* or summary).ti. or review.pt.)
17. (0266-4623 or 1469-493X or 1366-5278 or 1530-440X or 2046-4053).is.
18. meta-analysis.pt. or (meta-analys* or meta analys* or metaanalys* or meta synth* or meta-synth* or metasynth*).ti,ab,kf,hw.
19. ((systematic or meta) adj2 (analys* or review)).ti,kf. or ((systematic* or quantitativ* or methodologic*) adj5 (review* or overview*)).ti,ab,kf,sh. or (quantitativ$ adj5 synthesis$).ti,ab,kf,hw.
20. (integrative research review* or research integration).tw. or scoping review?.ti,kf. or (review.ti,kf,pt. and (trials as topic or studies as topic).hw.) or (evidence adj3 review*).ti,ab,kf.
21. review.pt. and ((medline or medlars or embase or pubmed or scisearch or psychinfo or psycinfo or psychlit or psyclit or cinahl or electronic database* or bibliographic database* or computeri#ed database* or online database* or pooling or pooled or mantel haenszel or peto or dersimonian or der simonian or fixed effect or ((hand adj2 search*) or (manual* adj2 search*))).tw,hw. or (retraction of publication or retracted publication).pt.)
22. or/16-21
23. 15 and 22

Body fatness - Insulin signalling RCT search

1. *body fat distribution/ or *adiposity/ or *body mass index/ or *body weight/ or *overweight/ or *obesity/ or *waist circumference/ or *waist-height ratio/ or *skinfold thickness/ or *waist-hip ratio/
2. exp *Adipose Tissue/

3. *body weight changes/ or *weight gain/ or *weight loss/
4. or/1-3
5. *Insulin/
6. *Insulin Resistance/
7. exp *Hyperinsulinism/
8. Glycated Hemoglobin A.ti,ab,kf.
9. or/5-8
10. 4 and 9
11. ((body weight changes or weight gain or weight loss or weight reduction) adj3 (insulin* or hyperinsulin*)).ti,ab,kf.
12. ((body weight changes or weight gain or weight loss or weight reduction) adj5 (fasting glucose or proinsulin or pro-insulin or c-peptide or HbA1c or h?emoglobin A1c or HOMA-IR or HOMA-S or HOMA or QUICKI)).ti,ab,kf.
13. 10 or 11 or 12
14. controlled clinical trial.pt.
15. randomized controlled trial.pt.
16. clinical trials as topic/
17. (randomi#ed or randomi#ation or randomi#ing).ti,ab,kf.
18. (RCT or "at random" or (random* adj3 (administ* or allocat* or assign* or class* or cluster or crossover or cross-over or control* or determine* or divide* or division or distribut* or expose* or fashion or number* or place* or pragmatic or quasi or recruit* or split or subsitut* or treat*))).ti,ab,kf.
19. placebo.ab,ti,kf.
20. trial.ti.
21. (control* adj3 group*).ab.
22. (control* and (trial or study or group*) and (waitlist* or wait* list* or ((treatment or care) adj2 usual))).ti,ab,kf,hw.
23. ((single or double or triple or treble) adj2 (blind* or mask* or dummy)).ti,ab,kf.
24. double-blind method/ or random allocation/ or single-blind method/
25. or/14-24
26. 13 and 25

Insulin signalling – PCa search (combined with the below systematic review and RCT study filters)

1. Insulin.af.
2. Exp Hyperinsulinism/
3. Hyperinsulin* .ti,ab,kf.
4. (proinsulin or pro-insulin or c-peptide).mp.
5. (HbA1c or h?emoglobinA1c or HOMA-IR or HOMA-S or QUICKI or fasting glucose).ti,ab,kf.
6. Glycated Hemoglobin A/me [Metabolism]
7. Or/1-6
8. exp Prostatic Neoplasms/
9. Prostatic Intraepithelial Neoplasia/
10. (prostat* adj3 (hyperplas* or neoplas* or cancer* or carcinoma* or adenocarcinoma* or sarcoma* or tumo?r* or metasta*)).ti,ab,kf.
11. prostat*.ti,kf.
12. or/8-11
13. 7 and 12
14. (insulin adj5 prostat*).ti,ab,kf
15. 13 or 14

Systematic review filter:

1. (systematic or structured or evidence or trials or studies).ti. and ((review or overview or look or examination or update* or summary).ti. or review.pt.)

2. (0266-4623 or 1469-493X or 1366-5278 or 1530-440X or 2046-4053).is.

3. meta-analysis.pt. or (meta-analys* or meta analys* or metaanalys* or meta synth* or meta-synth* or metasynth*).ti,ab,kf,hw.

4. ((systematic or meta) adj2 (analys* or review)).ti,kf. or ((systematic* or quantitativ* or methodologic*) adj5 (review* or overview*)).ti,ab,kf,sh. or (quantitativ$ adj5 synthesis$).ti,ab,kf,hw.

5. (integrative research review* or research integration).tw. or scoping review?.ti,kf. or (review.ti,kf,pt. and (trials as topic or studies as topic).hw.) or (evidence adj3 review*).ti,ab,kf.

6. review.pt. and ((medline or medlars or embase or pubmed or scisearch or psychinfo or psycinfo or psychlit or psyclit or cinahl or electronic database* or bibliographic database* or computeri#ed database* or online database* or pooling or pooled or mantel haenszel or peto or dersimonian or der simonian or fixed effect or ((hand adj2 search*) or (manual* adj2 search*))).tw,hw. or (retraction of publication or retracted publication).pt.)

7. or/1-6

RCT filter:

1. controlled clinical trial.pt.

2. randomized controlled trial.pt.

3. clinical trials as topic/

4. (randomi#ed or randomi#ation or randomi#ing).ti,ab,kf.

5. (RCT or "at random" or (random* adj3 (administ* or allocat* or assign* or class* or cluster or crossover or cross-over or control* or determine* or divide* or division or distribut* or expose* or fashion or number* or place* or pragmatic or quasi or recruit* or split or subsitut* or treat*))).ti,ab,kf.

6. placebo.ab,ti,kf.

7. trial.ti.

8. (control* adj3 group*).ab.

9. (control* and (trial or study or group*) and (waitlist* or wait* list* or ((treatment or care) adj2 usual))).ti,ab,kf,hw.

10. ((single or double or triple or treble) adj2 (blind* or mask* or dummy)).ti,ab,kf.

11. double-blind method/ or random allocation/ or single-blind method/

12. or/1-11

Embase search strategy

Body fatness - Insulin signalling systematic review search

24. *body fat distribution/ or *adiposity/ or *body mass index/ or *body weight/ or *overweight/ or *obesity/ or *waist circumference/ or *waist-height ratio/ or *skinfold thickness/ or *waist-hip ratio/
25. exp *Adipose Tissue/
26. *body weight changes/ or *weight gain/ or *weight loss/
27. or/1-3
28. *Insulin/
29. *Insulin Resistance/
30. exp *Hyperinsulinism/

31. *Glycated Hemoglobin A.ti,ab,kw.
32. or/5-8
33. 4 and 9
34. ((adiposity or body mass or BMI or fat or fatness or weight or overweight or over-weight or obese or obesity) adj3 (insulin* or hyperinsulin*)).ti,ab,kw.
35. ((adiposity or body mass or BMI or fat or fatness or weight or overweight or over-weight or obese or obesity) adj5 (fasting glucose or proinsulin or pro-insulin or c-peptide or HbA1c or h?emoglobin A1c or HOMA-IR or HOMA-S or HOMA or QUICKI)).ti,ab,kw.
36. (waist adj2 (hip or height) adj2 ratio? adj5 (insulin* or hyperinsulin*)).ti,ab,kw.
37. (waist adj2 (hip or height) adj2 ratio? adj5 (fasting glucose or proinsulin or pro-insulin or c-peptide or HbA1c or h?emoglobin A1c or HOMA-IR or HOMA-S or HOMA or QUICKI)).ti,ab,kw.
38. or/10-14
39. systematic review/ or meta analysis/ or network meta-analysis/
40. ((systematic or structured or evidence or trials or studies) and (review or overview or look or examination or update* or summary)).ti.
41. (0266-4623 or 1469-493X or 1366-5278 or 1530-440X or 2046-4053).is.
42. (systematic review? or evidence report* or technology assessment?).jw.
43. (meta-analys* or meta analys* or metaanalys* or meta synth* or meta-synth* or metasynth*).ti,ab,kw,hw.
44. ((systematic or meta) adj2 (analys* or review)).ti,kw. or ((systematic* or quantitativ* or methodologic*) adj5 (review* or overview*)).ti,ab,kw,sh. or (quantitativ* adj5 synthes*).ti,ab,kw,hw.
45. exp "clinical trial (topic)"/ and review.ti,kw,pt.
46. (integrative research review* or research integration).ti,ab,kw. or scoping review?.ti,kw. or (evidence adj3 review*).ti,ab,kw.
47. review.pt. and (medline or medlars or embase or pubmed or scisearch or psychinfo or psycinfo or psychlit or psyclit or cinahl or electronic database* or bibliographic database* or computeri#ed database* or online database* or pooling or pooled or mantel haenszel or peto or dersimonian or der simonian or fixed effect or ((hand adj2 search*) or (manual* adj2 search*))).ti,ab,kw,hw.
48. review.pt. and ((evidence based adj (medicine or practice)) or (outcome? adj (assessment or research)) or treatment outcome).hw.
49. or/16-25
50. 15 and 26

Body fatness - Insulin signalling RCT search

27. *body fat distribution/ or *adiposity/ or *body mass index/ or *body weight/ or *overweight/ or *obesity/ or *waist circumference/ or *waist-height ratio/ or *skinfold thickness/ or *waist-hip ratio/
28. exp *Adipose Tissue/
29. *body weight changes/ or *weight gain/ or *weight loss/
30. or/1-3
31. *Insulin/
32. *Insulin Resistance/
33. exp *Hyperinsulinism/
34. Glycated Hemoglobin A.ti,ab,kw.
35. or/5-8
36. 4 and 9
37. ((body weight changes or weight gain or weight loss or weight reduction) adj3 (insulin* or hyperinsulin*)).ti,ab,kw.
38. ((body weight changes or weight gain or weight loss or weight reduction) adj5 (fasting glucose or proinsulin or pro-insulin or c-peptide or HbA1c or h?emoglobin A1c or HOMA-IR or HOMA-S or HOMA or QUICKI)).ti,ab,kw.
39. 10 or 11 or 12
40. randomized controlled trial/
41. randomization.de.

42. controlled clinical trial/ and (Disease Management or Drug Therapy or Prevention or Rehabilitation or Therapy).fs.
43. *clinical trial/
44. placebo.de.
45. placebo.ti,ab.
46. trial.ti.
47. (randomi#ed or randomi#ation or randomi#ing).ti,ab,kw.
48. (RCT or "at random" or (random* adj3 (administ* or allocat* or assign* or class* or cluster or control* or crossover or cross-over or determine* or divide* or division or distribut* or expose* or fashion or number* or place* or pragmatic or quasi or recruit* or split or subsitut* or treat*))).ti,ab,kw.
49. ((singl$ or doubl$ or trebl$ or tripl$) adj3 (blind$ or mask$ or dummy)).mp.
50. (control* and (study or group?) and (waitlist* or wait* list* or ((treatment or care) adj2 usual))).ti,ab,kw,hw.
51. or/14-24
52. 13 and 25

Insulin signalling – PCa search (combined with the below systematic review and RCT study filters)

16. Insulin.af.
17. Exp Hyperinsulinism/
18. Hyperinsulin* .ti,ab,kw.
19. (proinsulin or pro-insulin or c-peptide).mp.
20. (HbA1c or h?emoglobinA1c or HOMA-IR or HOMA-S or QUICKI or fasting glucose).ti,ab,kw.
21. Glycated Hemoglobin A.mp.
22. Or/1-6
23. exp Prostatic Neoplasms/
24. Prostatic Intraepithelial Neoplasia/
25. (prostat* adj3 (hyperplas* or neoplas* or cancer* or carcinoma* or adenocarcinoma* or sarcoma* or tumo?r* or metasta*)).ti,ab,kw.
26. prostat*.ti,kw.
27. or/8-11
28. 7 and 12
29. (insulin adj5 prostat*).ti,ab,kw.
30. 13 or 14

Systematic review filter:

1. systematic review/ or meta analysis/ or network meta-analysis/
2. ((systematic or structured or evidence or trials or studies) and (review or overview or look or examination or update* or summary)).ti.
3. (0266-4623 or 1469-493X or 1366-5278 or 1530-440X or 2046-4053).is.
4. (systematic review? or evidence report* or technology assessment?).jw.
5. (meta-analys* or meta analys* or metaanalys* or meta synth* or meta-synth* or metasynth*).ti,ab,kw,hw.
6. ((systematic or meta) adj2 (analys* or review)).ti,kw. or ((systematic* or quantitativ* or methodologic*) adj5 (review* or overview*)).ti,ab,kw,sh. or (quantitativ* adj5 synthes*).ti,ab,kw,hw.
7. exp "clinical trial (topic)"/ and review.ti,kw,pt.
8. (integrative research review* or research integration).ti,ab,kw. or scoping review?.ti,kw. or (evidence adj3 review*).ti,ab,kw.
9. review.pt. and (medline or medlars or embase or pubmed or scisearch or psychinfo or psycinfo or psychlit or psyclit or cinahl or electronic database* or bibliographic database* or computeri#ed database* or online database* or pooling or pooled or mantel haenszel or peto or dersimonian or der simonian or fixed effect or ((hand adj2 search*) or (manual* adj2 search*))).ti,ab,kw,hw.
10. review.pt. and ((evidence based adj (medicine or practice)) or (outcome? adj (assessment or research)) or treatment outcome).hw.

11. or/1-10

RCT filter:

1. randomized controlled trial/
2. randomization.de.
3. controlled clinical trial/ and (Disease Management or Drug Therapy or Prevention or Rehabilitation or Therapy).fs.
4. *clinical trial/
5. placebo.de.
6. placebo.ti,ab.
7. trial.ti.
8. (randomi#ed or randomi#ation or randomi#ing).ti,ab,kw.
9. (RCT or "at random" or (random* adj3 (administ* or allocat* or assign* or class* or cluster or control* or crossover or cross-over or determine* or divide* or division or distribut* or expose* or fashion or number* or place* or pragmatic or quasi or recruit* or split or subsitut* or treat*))).ti,ab,kw.
10. ((singl$ or doubl$ or trebl$ or tripl$) adj3 (blind$ or mask$ or dummy)).mp.
11. (control* and (study or group?) and (waitlist* or wait* list* or ((treatment or care) adj2 usual))).ti,ab,kw,hw.
12. or/1-11

BIOSIS search strategy

Body fatness - Insulin signalling systematic review search

1. TS = (body fat distribution or adiposity or body mass index or body weight or overweight or obesity or waist circumference or waist-height ratio or skinfold thickness or waist-hip ratio)
2. TS = adipose tissue
3. TS = (body weight changes or weight gain or weight loss)
4. #1 OR #2 OR #3
5. TS = (insulin or insulin resistance or hyperinsulin*)
6. TS = Glycated Hemoglobin A
7. #5 OR #6
8. #4 AND #7
9. TS = ((adiposity near/3 (insulin* or hyperinsulin*)) or (body mass near/3 (insulin* or hyperinsulin*)) or (BMI near/3 (insulin* or hyperinsulin*)) or (fat near/3 (insulin* or hyperinsulin*)) or (fatness near/3 (insulin* or hyperinsulin*)) or (weight near/3 (insulin* or hyperinsulin*)) or (overweight near/3 (insulin* or hyperinsulin*)) or (over-weight near/3 (insulin* or hyperinsulin*)) or (obese near/3 (insulin* or hyperinsulin*)) or (obesity near/3 (insulin* or hyperinsulin*)))
10. TS = (waist near/2 (hip or height) near/2 ratio? near/5 (insulin* or hyperinsulin*))
11. TS=(waist near/2 (hip or height) near/2 ratio? near/5 (proinsulin or pro-insulin or c-peptide or HbA1c or HOMA-IR or HOMA-S or HOMA or QUICKI or fasting glucose or h$emoglobin A1c))
12. TS = ((adiposity near/5 (proinsulin or pro-insulin or c-peptide or HbA1c or HOMA-IR or HOMA-S or HOMA or QUICKI or fasting glucose or h$emoglobin A1c)) or (body mass near/5 (proinsulin or pro-insulin or c-peptide or HbA1c or HOMA-IR or HOMA-S or HOMA or QUICKI or fasting glucose or h$emoglobin A1c)) or (BMI near/5 (proinsulin or pro-insulin or c-peptide or HbA1c or HOMA-IR or HOMA-S or HOMA or QUICKI or fasting glucose or h$emoglobin A1c)) or (fat near/5 (proinsulin or pro-insulin or c-peptide or HbA1c or HOMA-IR or HOMA-S or HOMA or QUICKI or fasting glucose or h$emoglobin A1c)) or (fatness near/5 (proinsulin or pro-insulin or c-peptide or HbA1c or HOMA-IR or HOMA-S or HOMA or QUICKI or fasting glucose or h$emoglobin A1c)) or (weight near/5 (proinsulin or pro-insulin or c-peptide or HbA1c or HOMA-IR or HOMA-S or HOMA or QUICKI or fasting glucose or h$emoglobin A1c)) or (overweight near/5 (proinsulin or pro-insulin or c-peptide or HbA1c or HOMA-IR or HOMA-S or HOMA or QUICKI or fasting glucose or h$emoglobin A1c)) or (over-weight near/5 (proinsulin or pro-insulin or c-peptide or HbA1c or HOMA-IR or HOMA-S or HOMA or QUICKI or fasting glucose or h$emoglobin A1c)) or (obese near/5 (proinsulin or pro-insulin or c-peptide or HbA1c or HOMA-IR or HOMA-S or HOMA or QUICKI or fasting glucose or h$emoglobin A1c)) or (obesity near/5 (proinsulin or pro-insulin or c-peptide or HbA1c or HOMA-IR or HOMA-S or HOMA or QUICKI or fasting glucose or h$emoglobin A1c)))
13. #8 OR #9 OR #10 OR #11 OR #12
14. (TI=((systematic or structured or evidence or trials or studies) and (review or overview or look or examination or update* or summary))) OR (TI=((systematic or meta) SAME (analys* or review))) OR (TI=(scoping and review)) OR (TS=(meta-analys* or "meta analys*" or metaanalys* or "meta synth*" or meta-synth* or metasynth*)) OR (TS=((systematic* or quantitativ* or methodologic*) SAME (review* or overview*))) OR (TS=(“integrative research review*” or “research integration” OR “literature review”)) OR (TS=(evidence SAME review*)) OR (TS=review AND TS=(search* SAME (medline or medlars or embase or pubmed or scisearch or psychinfo or psycinfo or psychlit or psyclit or cinahl or biosis or "web of science" or "electronic database*" or "bibliographic database*" or "computerized database*" or "computerised database*" or "online database*" or "trial register*")))
15. 13 and 14

Body fatness - Insulin signalling RCT search

1. TS = (body weight changes or weight gain or weight loss)

2. TS = (insulin or insulin resistance or hyperinsulin*)
3. TS = Glycated Hemoglobin A
4. #2 OR #3
5. #4 AND #1
6. TS = ((body weight changes near/3 (insulin* or hyperinsulin*)) or (weight gain near/3 (insulin* or hyperinsulin*)) or (weight loss near/3 (insulin* or hyperinsulin*)) or (weight reduction near/3 (insulin* or hyperinsulin*)))
7. TS = ((body weight changes near/5 (proinsulin or pro-insulin or c-peptide or HbA1c or HOMA-IR or HOMA-S or HOMA or QUICKI or fasting glucose or h$emoglobin A1c)) or (weight gain near/5 (proinsulin or pro-insulin or c-peptide or HbA1c or HOMA-IR or HOMA-S or HOMA or QUICKI or fasting glucose or h$emoglobin A1c)) or (weight loss near/5 (proinsulin or pro-insulin or c-peptide or HbA1c or HOMA-IR or HOMA-S or HOMA or QUICKI or fasting glucose or h$emoglobin A1c)) or (weight reduction near/5 (proinsulin or pro-insulin or c-peptide or HbA1c or HOMA-IR or HOMA-S or HOMA or QUICKI or fasting glucose or h$emoglobin A1c)))
8. #5 OR #6 OR #7
9. TS=(randomised OR randomized OR randomisation OR randomisation OR placebo* OR (random* AND (allocat* OR assign*)) OR (blind* AND (single OR double OR treble OR triple)))
10. 8 and 9

Insulin signalling – PCa search (combined with the below systematic review and RCT study filters)

1. TS = (insulin)
2. TS = (hyperinsulin*)
3. TS = (insulin resistance)
4. TS = (insulin NEAR/2 (blood* or serum or plasma))
5. TS = (insulin signalling or insulin receptor)
6. TS = (proinsulin or pro-insulin or c-peptide)
7. TS = (HbA1c or h?emoglobin A1c or HOMA-IR or HOMA-S or HOMA or QUICKI or fasting glucose)
8. TS = (glycated Hemoglobin A)
9. #1 OR #2 OR #3 OR #4 OR #5 OR #6 OR #7 OR #8
10. TS = (prostat* neoplas* or prostat* cancer or prostat* carcinoma or prostat* tumo$r)
11. TS = (prostatic intraepithelial neoplasia)
12. #10 OR #11
13. TS = (neoplasm metastasis or neoplasm invasiveness)
14. #13 AND #10
15. #14 OR #12

Systematic review filter:

(TI=(evidence or review or overview or look or examination or update* or summary)) OR (TS=((systematic or meta) SAME (analys* or review))) OR (TS=(scoping SAME review)) OR (TS=(meta-analys* or "meta analys*" or metaanalys* or "meta synth*" or meta-synth* or metasynth*)) OR (TS=((systematic* or quantitativ* or methodologic*) SAME (review* or overview*))) OR (TS=(“integrative research review*” or “research integration” OR “literature review”)) OR (TS=(evidence AND review*)) OR (TS=("we review*" or "this review")) OR (TS=review AND TS=(search* SAME (medline or medlars or embase or pubmed or scisearch or psychinfo or psycinfo or psychlit or psyclit or cinahl or biosis or "web of science" or "electronic database*" or "bibliographic database*" or "computerized database*" or "computerised database*" or "online database*" or "trial register*")))

RCT filter:

TS=(randomised OR randomized OR randomisation OR randomisation OR placebo* OR (ra ndom* AND (allocat* OR assign*)) OR (blind* AND (single OR double OR treble OR triple)))

Table S7: Prostate cancer outcome categories by study

Outcome category used for this review	Outcomes included in the original study	Studies
PCa total	PCa total	Albanes D (2009), Dickerman BA (2018), Marrone MT (2019), Lai GY (2010), Lai GY (2014), Darbinian JA (2008)
PCa, localised	PCa, localised (T1–T2, N0, M0)	Lai GY (2010)
	PCa, localized (T1b-T2c and N0M0)	Lai GY (2014)
	PCa, localised (stage 1)	Darbinian JA (2008)
	PCa (Stage: 0-II)	Albanes D (2009)
PCa, advanced	PCa (Stage: III-IV)	Albanes D (2009)
	PCa, advanced (clinical stage >T3b or N1 or M1 at diagnosis, or died of prostate cancer during follow-up)	Lai GY (2014), Dickerman BA (2018)
	PCa, advanced (T3, T4, N1, M1 or fatal)	Lai GY (2010)

	Lethal Prostate Cancer Incidence: A first primary prostate cancer case that either had distant metastasis to any organ at diagnosis (pathologic TNM stage 4 or SEER summary stage 3, 4, or 7) or that led to death with prostate cancer as the underlying cause	Marrone MT (2019)
PCa, low-grade (Gleason sum <7)	PCa, low-grade (Gleason sum <7)	Lai GY (2010), Lai GY (2014)
PCa, high-grade (Gleason sum ≥7)	PCa, high-grade (Gleason sum ≥7)	Lai GY (2010), Lai GY (2014)
	PCa, high-grade (Gleason score 8–10)	Dickerman BA (2018)
PCa mortality	PCa mortality	Dickerman BA (2018), Marrone MT (2019)
PCa, regional (stages 2-5), distant (stage 7)	PCa, regional (stages 2-5), distant (stage 7)	Darbinian JA (2008)

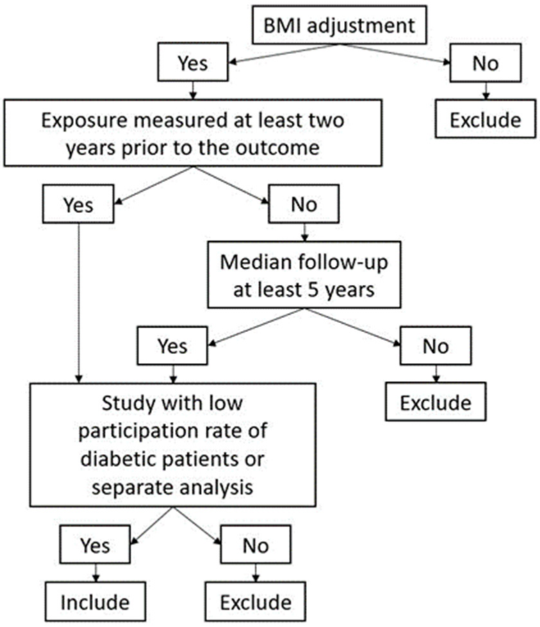

Figure S1 - Inclusion/exclusion criteria applied to potentially eligible insulin-prostate cancer studies

References

1. Alsubheen SA, Ismail M, Baker A, et al. The effects of diurnal Ramadan fasting on energy expenditure and substrate oxidation in healthy men. Br J Nutr. 2017;118(12):1023-1030. doi:10.1017/S0007114517003221

2. Dengel DR, Galecki AT, Hagberg JM, Pratley RE. The independent and combined effects of weight loss and aerobic exercise on blood pressure and oral glucose tolerance in older men. Am J Hypertens. 1998;11(12):1405-1412. doi:10.1016/S0895-7061(98)00185-X

3. McAllister MJ, Pigg BL, Renteria LI, Waldman HS. Time-restricted feeding improves markers of cardiometabolic health in physically active college-age men: a 4-week randomized pre-post pilot study. Nutr Res. 2020;75:32-43. doi:10.1016/j.nutres.2019.12.001

4. Kaukua J, Pekkarinen T, Sane T, Mustajoki P. Sex hormones and sexual function in obese men losing weight. Obes Res. 2003;11(6):689-694. doi:10.1038/oby.2003.98

5. Johnson ML, Distelmaier K, Lanza IR, et al. Mechanism by which caloric restriction improves insulin sensitivity in sedentary obese adults. Diabetes. 2016;65(1):74-84. doi:10.2337/db15-0675

6. Wright JL, Plymate S, D'Oria-Cameron A, et al. A study of caloric restriction versus standard diet in overweight men with newly diagnosed prostate cancer: A randomized controlled trial. Prostate. 2013;73(12):1345-1351. doi:10.1002/pros.22682

7. Chan DC, Watts GF, Ng TWK, Yamashita S, Barrett PHR. Effect of weight loss on markers of triglyceride-rich lipoprotein metabolism in the metabolic syndrome. Eur J Clin Invest. 2008;38(10):743-751. doi:10.1111/j.1365-2362.2008.02019.x

8. Jacobs DR, Sluik D, Rokling-Andersen MH, Anderssen SA, Drevon CA. Association of 1-y changes in diet pattern with cardiovascular disease risk factors and adipokines: Results from the 1-y randomized oslo diet and exercise study. Am J Clin Nutr. 2009;89(2):509-517. doi:10.3945/ajcn.2008.26371

9. Moro T, Tinsley G, Bianco A, et al. Effects of eight weeks of time-restricted feeding (16/8) on basal metabolism, maximal strength, body composition, inflammation, and cardiovascular risk factors in resistance-trained males. J Transl Med. 2016;14(1):1-10. doi:10.1186/s12967-016-1044-0

10. Ng TWK, Chan DC, Barrett PHR, Watts GF. Effect of weight loss on HDL-apoA-II kinetics in the metabolic syndrome. Clin Sci. 2010;118(1):79-85. doi:10.1042/CS20090110

11. Tanaka S, Uenishi K, Ishida H, et al. A randomized intervention trial of 24-wk dairy consumption on waist circumference, blood pressure, and fasting blood sugar and lipids in Japanese men with metabolic syndrome. J Nutr Sci Vitaminol (Tokyo). 2014;60(5):305-312. doi:10.3177/jnsv.60.305

12. Stocks T, Lukanova A, Rinaldi S, et al. Insulin resistance is inversely related to prostate cancer: A prospective study in Northern Sweden. Int J Cancer. 2007;120(12):2678-2686. doi:10.1002/ijc.22587

13. Arthur R, Møller H, Garmo H, et al. Serum glucose, triglycerides, and cholesterol in relation to prostate cancer death in the Swedish AMORIS study. Cancer Causes Control. 2019;30(2):195-206. doi:10.1007/s10552-018-1093-1

14. Inoue M, Noda M, Kurahashi N, et al. Impact of metabolic factors on subsequent cancer risk: Results from a large-scale population-based cohort study in Japan. Eur J Cancer Prev. 2009;18(3):240-247. doi:10.1097/CEJ.0b013e3283240460

15. Hubbard JS, Rohrmann S, Landis PK, et al. Association of prostate cancer risk with insulin, glucose, and anthropometry in the baltimore longitudinal study of aging. Urology. 2004;63(2):253-258. doi:10.1016/j.urology.2003.09.060

16. Travier N, Jeffreys M, Brewer N, et al. Association between glycosylated hemoglobin and cancer risk: A New Zealand linkage study. Ann Oncol. 2007;18(8):1414-1419. doi:10.1093/annonc/mdm135

17. Tande AJ, Platz EA, Folsom AR. The metabolic syndrome is associated with reduced risk of prostate cancer. Am J Epidemiol. 2006;164(11):1094-1102. doi:10.1093/aje/kwj320

18. Murtola TJ, Vihervuori VJY, Lahtela J, et al. Fasting blood glucose, glycaemic control and prostate cancer risk in the Finnish Randomized Study of Screening for Prostate Cancer. Br J Cancer. 2018;118(9):1248-1254. doi:10.1038/s41416-018-0055-4

19. Jee SH, Ohrr H, Sull JW, Yun J, Ji M, Samet J. Fasting Serum Glucose Level and Cancer Risk in Korean Men and Women. JAMA. 2005;293(2):194. doi:10.1001/jama.293.2.194

20. Murtola TJ, Sälli SM, Talala K, Taari K, Tammela TLJ, Auvinen A. Blood glucose, glucose balance, and disease-specific survival after prostate cancer diagnosis in the Finnish Randomized Study of Screening for Prostate Cancer. Prostate Cancer Prostatic Dis.

2019;22(3):453-460. doi:10.1038/s41391-018-0123-0

21. Kiyabu GY, Sawada N, Iwasaki M, et al. The association between plasma C-peptide concentration and the risk of prostate cancer: A nested case-control study within a Japanese population-based prospective study. Eur J Cancer Prev. 2018;27(5):461-467. doi:10.1097/CEJ.0000000000000363

22. Stattin P, Bylund A, Rinaldi S, et al. Plasma Insulin-Like Growth Factor-I, Insulin-Like Growth Factor-Binding Proteins, and Prostate Cancer Risk: a Prospective Study. J Natl Cancer Inst. 2000;92(23):1910-1917. doi:10.1093/jnci/92.23.1910

23. Stevens VL, Jacobs EJ, Sun J, Gapstur SM. No association of plasma levels of adiponectin and c-peptide with risk of aggressive prostate cancer in the cancer prevention study II nutrition cohort. Cancer Epidemiol Biomarkers Prev. 2014;23(5):890-892. doi:10.1158/1055-9965.EPI-14-0114

24. Goto A, Noda M, Sawada N, et al. High hemoglobin A1c levels within the non-diabetic range are associated with the risk of all cancers. Int J Cancer. 2016;138(7):1741-1753. doi:10.1002/ijc.29917

25. Joshu CE, Prizment AE, Dluzniewski PJ, et al. Glycated hemoglobin and cancer incidence and mortality in the Atherosclerosis in Communities (ARIC) Study, 1990-2006. Int J Cancer. 2012;131(7):1667-1677. doi:10.1002/ijc.27394

26. Kim SH, Kim S, Joung JY, et al. Lifestyle risk prediction model for prostate cancer in a Korean population. Cancer Res Treat. 2018;50(4):1194-1202. doi:10.4143/crt.2017.484

27. Parekh N, Lin Y, Vadiveloo M, Hayes RB, Lu-Yao GL. Metabolic dysregulation of the insulin-glucose axis and risk of obesity-related cancers in the Framingham heart study-offspring cohort (1971-2008). Cancer Epidemiol Biomarkers Prev. 2013;22(10):1825-1836. doi:10.1158/1055-9965.EPI-13-0330

28. Grundmark B, Garmo H, Loda M, Busch C, Holmberg L, Zethelius B. The Metabolic Syndrome and the Risk of Prostate Cancer under Competing Risks of Death from Other Causes. Cancer Epidemiol Biomarkers Prev. 2010;19(8):2088-2096. doi:10.1158/1055-9965.EPI-10-0112

29. Nguyen MM, Martinez JA, Hsu C-H, et al. Bioactivity and prostate tissue distribution of metformin in a preprostatectomy prostate cancer cohort. Eur J Cancer Prev. 2018;27(6):557-562. doi:10.1097/CEJ.0000000000000394
